# Oral ondansetron versus domperidone for symptomatic treatment of vomiting during acute gastroenteritis in children: multicentre randomized controlled trial

**DOI:** 10.1186/1471-2431-11-15

**Published:** 2011-02-10

**Authors:** Federico Marchetti, Alessandra Maestro, Francesca Rovere, Davide Zanon, Alberto Arrighini, Paolo Bertolani, Paolo Biban, Liviana Da Dalt, Pasquale Di Pietro, Salvatore Renna, Andrea Guala, Francesco Mannelli, Anna Pazzaglia, Gianni Messi, Francesco Perri, Antonino Reale, Antonio Francesco Urbino, Enrico Valletta, Antonio Vitale, Tiziana Zangardi, Maria Teresa Tondelli, Antonio Clavenna, Maurizio Bonati, Luca Ronfani

**Affiliations:** 1 Department of Pediatrics and Epidemiology and Biostatistics Unit, Institute for Maternal and Child Health, IRCCS Burlo Garofolo, Trieste, Italy; 2 Clinical Services of Pharmacy, Institute for Maternal and Child Health, IRCCS Burlo Garofolo, Trieste, Italy; 3 Pediatric Emergency Department, P.O. Spedali Civili, Brescia, Italy; 4 Department of Pediatrics, Azienda Policlinico, Modena, Italy; 5 Pediatric Intensive Care Unit, Ospedale Civile Maggiore, Verona, Italy; 6 Department of Pediatrics, Ospedale di Treviso, Treviso, Italy; 7 Emergency Room and Emergency Medicine Division, G. Gaslini Institute, Genova, Italy; 8 Department of Pediatrics, Ospedale Castelli, Verbania, Italy; 9 Emergency Department, Paediatric Hospital A. Meyer, Firenze, Italy; 10 Emergency Department, Institute for Maternal and Child Health - IRCCS Burlo Garofolo, Trieste, Italy; 11 Department of Pediatrics, Ospedale di Macerata, Macerata, Italy; 12 Emergency Department, Ospedale Pediatrico Bambino Gesú, IRCCS, Roma, Italy; 13 Emergency Department, Ospedale Infantile Regina Margherita di Torino, Torino, Italy; 14 Department of Pediatrics, Ospedale GB Morgagni, Forlì, Italy; 15 Pediatric Emergency Department, Ospedale Giuseppe Moscati, Avellino, Italy; 16 Pediatric Emergency Department, Azienda Ospedaliera - University of Padova, Padova, Italy; 17 Department of Pediatrics, Azienda Ospedaliera, University of Parma, Italy; 18 Maternal and Child Health Laboratory, Institute Mario Negri, Milano, Italy

## Abstract

**Background:**

Vomiting in children with acute gastroenteritis (AG) is not only a direct cause of fluid loss but it is also a major factor of failure of oral rehydration therapy (ORT). Physicians who provide care to paediatric patients in the emergency department (ED) usually prescribe intravenous fluid therapy (IVT) for mild or moderate dehydration when vomiting is the major symptom. Thus, effective symptomatic treatment of vomiting would lead to an important reduction in the use of IVT and, consequently, of the duration of hospital stay and of frequency of hospital admission. Available evidence on symptomatic treatment of vomiting shows the efficacy of the most recently registered molecule (ondansetron) but a proper evaluation of antiemetics drugs largely used in clinical practice, such as domperidone, is lacking.

**Objectives:**

To compare the efficacy of ondansetron and domperidone for the symptomatic treatment of vomiting in children with AG who have failed ORT.

**Methods/Design:**

Multicentre, double-blind randomized controlled trial conducted in paediatric EDs. Children aged from 1 to 6 years who vomiting, with a presumptive clinical diagnosis of AG, and without severe dehydration will be included. After the failure of a initial ORS administration in ED, eligible children will be randomized to receive: 1) ondansetron syrup (0,15 mg/Kg of body weight); 2) domperidone syrup (0,5 mg/Kg of body weight); 3) placebo. The main study outcome will be the percentage of patients needing nasogastric or IVT after symptomatic oral treatment failure, defined as vomiting or fluid refusal after a second attempt of ORT. Data relative to study outcomes will be collected at 30 minute intervals for a minimum of 6 hours. A telephone follow up call will be made 48 hours after discharge. A total number of 540 children (i.e. 180 patients in each arm) will be enrolled.

**Discussion:**

The trial results would provide evidence on the efficacy of domperidone, which is largely used in clinical practice despite the lack of proper evaluation and a controversial safety profile, as compared to ondansetron, which is not yet authorized in Italy despite evidence supporting its efficacy in treating vomiting. The trial results would contribute to a reduction in the use of IVT and, consequently, in hospital admissions in children with AG. The design of this RCT, which closely reflect current clinical practice in EDs, will allow immediate transferability of results.

**Trial Registration:**

ClinicalTrials.gov: NCT01257672

## Background

### Acute Gastroenteritis

Acute gastroenteritis (AG) is the main cause of acute vomiting in children aged under 3 years and one of the most important reasons for access to the emergency department (ED) and admission to hospital.

In USA 1.5 millions of children under 5 years are diagnosed AG annually and 13% of these children are admitted to the hospital [1]. In Italy "esophagitis, gastroenteritis and a miscellaneous of digestive apparatus diseases" results to be the most important cause of hospital admission in paediatric age [2].

The World Health Organization (WHO), the American Academy of Pediatrics (AAP), and the European Society for Paediatric Gastroenterology, Hepatology, and Nutrition (ESPGHAN) working group and the Cochrane Library database recommend oral rehydration therapy (ORT) and prompt realimentation for mild to moderate gastroenteritis [3,4].

### Vomiting in gastroenteritis and need for pharmacological treatment

In the initial phase of viral AG, vomiting is a typical symptom [5]. Thus, in AG caused by rotavirus infection during the first 1 to 3 days, repeated vomiting is present in 75% of children [6]. Current practice recommendations for paediatric AG do not include pharmacologic treatment for vomiting [3]. However, vomiting from AG is distressing for patients and their families. In addition, vomiting is not only a direct cause of fluid loss but can also hamper successful oral rehydration therapy and it is a major factor of failure of ORT. Many physicians believe that vomiting is a contraindication to ORT. Physicians who provide care to paediatric patients in the emergency department consistently favour intravenous fluid therapy (IVT) for mild or moderate dehydration when vomiting is the major symptom [7,8]. Thus, effective treatment of vomiting would lead to an important reduction in the use of IVT. Antiemetic agents and their use in clinical practice. Various antiemetic agents have been used to prevent or reduce vomiting in children with gastroenteritis [9]. The phenothiazines are dopamine antagonists and act centrally by blocking the chemoreceptor trigger zone. They are used to prevent or treat vomiting associated with drugs such as opiates, general anaesthetics, and cytotoxics. Unfortunately, severe dystonic reactions sometimes occur with phenothiazines, especially in children.

Metoclopramide is a chlorinated procainamide derivative that acts primarily as a dopamine D2 receptor antagonist and has both central and peripheral actions. Metoclopramide also acts directly on the gastro-intestinal tract and it may be more effective than the phenothiazines for vomiting associated with gastro-duodenal disease. As with the phenothiazines, metoclopramide can induce acute dystonic reactions involving facial and skeletal muscle spasms and oculogyric crises. These dystonic effects are more common in paediatric age. In Italy metoclopramide use in children under 16 years old is not recommended because severe extrapyramidal reactions [10] were reported in case-control studies and case reports. Domperidone is a D2 receptor antagonist that acts on the chemoreceptor trigger zone. The medication also accelerates gastric emptying. Ondansetron is a specific 5HT3 antagonists which block 5HT3 receptors in the gastro-intestinal tract and in the central nervous system. It has been shown to be effective in the treatment of vomiting in patients receiving cytotoxic agents for cancer. Dexamethasone also has anti-emetic effects and is used to prevent vomiting associated with cancer chemotherapy. In this context it may be used alone or with other anti-emetics such as metoclopramide or a 5HT3 antagonist.

In the clinical practice antiemetic drugs are frequently used in children with gastroenteritis. A recent retrospective survey retrieved data from 4 national and international databases showed that prescription of antiemetic medication varied considerably [11]. In particular, between 2% and 23% of children with gastroenteritis received prescriptions for antiemetic medications. The antihistamines dimenhydrinate and diphenhydramine were most frequently used in Germany and Canada, whereas promethazine was prescribed preferentially in the United States. In France, Spain, and Italy, the dopamine receptor antagonist domperidone was preferred as antiemetic treatment. Ondansetron was used in a minor proportion of antiemetic prescriptions. A recent survey carried out in Italy showed that 79% of participating clinicians prescribe antiemetic drugs to treat acute gastroenteritis (domperidone in primis followed by metoclopramide) [12]. Data on Italian prescriptions collected by ARNO confirmed that among gastrointestinal agents, prokinetics (in 80% of cases domperidone) are the most prescribed in clinical practice [13].

### Antiemetic drugs in acute gastroenteritis: evidence of efficacy

As demonstrated in 3 recently published meta-analysis, literature evaluating the efficacy of symptomatic drugs in reducing acute vomiting for AG in paediatric age is methodologically limited and focuses mainly on ondansetron [14-16]. The 11 articles meeting the inclusion criteria evaluated various antiemetic agents [16]: ondansetron (n = 6), domperidone (n = 2), metoclopramide (n = 2), trimethobenzamide (n = 2), pyrilamine-pentobarbital (n = 2), dexamethasone (n = 1), and promethazine (n = 1). Six randomised controlled trials (RCTs) were carried out to evaluate ondansetron use in a total population of 745 children [17-22]. All these studies compared ondansetron versus placebo. Furthermore two RCTs included the comparison of ondansetron to metoclopramide and dexamethasone [16]. In three studies ondansetron was administrated per os and in the other three intravenous administration was preferred. Ondansetron, compared to placebo reduces the risk of future vomiting episodes (RR: 0.45; 95% CI: 0.33-0.62; Number Need to Treat, NNT = 5), the number of patients needing intravenous rehydration (RR, 0.41; 95% CI: 0.28 0.62; NNT = 5) and hospital admissions (RR, 0.52; 95% CI: 0.27-0.95; NNT = 14) [16]. The drug is not effective in reducing the access to ED for acute vomiting [14-16]. With respect to side effects, except for the greater incidence of diarrhoea in patients receiving ondansetron treatment [16], no other significant differences no other significant differences between ondansetron and placebo were identified.

A few studies were published regarding domperidone [23,24] and metoclopramide [21,23] and were characterized by small sample sizes, low methodological quality, and produced inconsistent results. These methodological issues do not allow to draw any conclusions about the risks/benefits balance of the two drugs [16]. Furthermore, an adequate comparative evaluation between domperidone, metoclopramide and ondansetron is missing. All the studies included in the analysis were funded by pharmaceutical companies.

### The need for further evidence and potential transferability to clinical practice

The above reported evidence shows the efficacy of the most recently registered molecule (ondansetron), but the studies were carried out on very heterogeneous populations for which risks, costs and benefits were not sufficiently assessed. The same evidence showed that a proper evaluation of anti-emetics drugs largely used in clinical practice [13], such as domperidone, is completely lacking. This lack of knowledge about antiemetics is particularly important considering the restricted indications for their use in Italy (this is particularly true for metoclopramide). In fact, these agents' potential side effects as extra-pyramidal manifestations, lack of consciousness, convulsions are mostly dose-dependent and they have been addressed by ad hoc report by Italian Agency of Drug (AIFA) paediatric commission [25].

In light of the above considerations, we propose a multicentre study comparing the efficacy of ondansetron and domperidone for the symptomatic treatment of vomiting in acute gastroenteritis. The study aims at answering the following clinical questions: a) would anti-emetics agents reduce the percentage of children who keep vomiting? b) would anti-emetics treatment favour the oral rehydration and reduce the need for nasogastric or intravenous fluid rehydration? c) would the treatment reduce the percentage of children accessing health services and needing hospital admission?

We believe that the results of such a trial could significantly impact current clinical practice. In fact it would define the real efficacy of domperidone largely used in clinical practice (despite the lack of a clear evidence-based assessment and a controversial safeness profile) compared to ondansetron whose use to treat vomiting in AG is not yet authorized in Italy despite evidence supporting its possible use.

## Objectives of the study

### Primary Objective

To evaluate whether the oral administration of a symptomatic drug (ondansetron or domperidone) prevents intravenous or nasogastric rehydration in children vomiting and diagnosed acute gastroenteritis.

### Secondary Objective

To assess whether the oral administration of a symptomatic drug (ondansetron or domperidone) reduces the total duration and number of vomiting episodes and the need for hospital admission or ED access.

## Methods/Design

### Study Design

The study is a prospective, multicentre, double-blind randomized controlled trial. The study will be coordinated by the Institute for Maternal and Child Health - IRCCS Burlo Garofolo (Trieste) and by the Maternal and Child Health Laboratory of the Institute Mario Negri (Milan).

### Ethical approval

Multi-centre approval has been granted by the Bioethics Committee of the Coordinating centre (Institute for Maternal and Child Health IRCCS Burlo Garofolo, Trieste), on November 8, 2010 (registration number: S-115). Site-specific approval has been granted by local Ethic Committee at all trial sites.

### Study Population

The study will be conducted in hospital and the recruitment setting will be the paediatric emergency departments (ED). Children between 1 to 6 years will be included in the study. Consecutive subjects accessing the ED during a 18 months period will be evaluated for inclusion/exclusion criteria by the doctor on call. The following inclusion and exclusion criteria will be used

### Inclusion Criteria

1) Age from 1 to 6 years;

2) presumptive clinical diagnosis of acute gastroenteritis in patients with vomiting, with or without diarrhoea (Table 1);

**Table 1:** Definitions of clinical diagnosis of acute gastroenteritis, vomiting, and standard protocol of oral rehydration solution administration

Clinical diagnosis of acute gastroenteritis
We refer to the NICE guideline (http://www.nice.org.uk/guidance/index.jsp?action=download&o=42316) that recommends:
*When considering a diagnosis of gastroenteritis, look for the following key characteristics:*
A recent change in stool consistency to loose or watery stools; recent onset of vomiting; recent contact with an individual with acute diarrhoea; exposure to known source of enteric infection (water or food borne); recent foreign travel.
*Consider the following symptoms and signs as possible indicators of diagnoses other than gastroenteritis:*
High fever (age less than 3 months: >38°C; age more than 3 months: >39°C); rapid breathing or labored respirations; altered conscious level (irritability, drowsiness); photophobia, neck stiffness and/or bulging fontanelle (in infants); non-blanching (haemorrhagic) rash; blood and/or mucous in stool; bilious vomiting (green); severe or localized abdominal pain; abdominal distension or rebound tenderness."

**Definition of Vomiting**

According to NICE, we define vomiting as the forceful ejection of the stomach contents up to and out of the mouth (http://www.nice.org.uk/guidance/index.jsp?action=download&o=42316).
Episodes separated by no more than two minutes are counted as a single episode. Non-productive retching, spilling of oral contents, and drooling were not considered vomiting.

**Standard protocol of oral rehydration solution (ORS) administration**

The following standard protocol is the result of the combination of international guidelines recommendations and study committee consensus derived from ED clinical practice:
*1st hour:*
-age 1 to 2 years: 50 cc of low osmolarity ORS (sodium 60 mmol/L) administered cold and in small, frequent volumes (small sips, time divided); this amount correspond to 1/2 coffee spoon (equivalent to 1,5 cc) every 2 minutes;
-age 3 to 6 years: 100 cc of low osmolarity ORS (sodium 60 mmol/L) administered cold and in small, frequent volumes (small sips, time divided); this amount correspond to 1 coffee spoon (equivalent to 3-3,5 cc) every 2 minutes.
*From 2nd to 6th hour:*
Cold ORS administered at sips following the plan:
- Mild dehydration: 30-60 ml/Kg body weight over 4-6 hours
- Moderate dehydration: 60-90 ml/Kg body weight over 4-6 hours

3) more than three episodes of non-bilious, non-bloody vomiting within the previous 24 hours;

### Exclusion Criteria

1) Treatment with antiemetics or antidiarrhoic drugs in the 6 hours prior to access to ED;

2) underlying chronic diseases (eg, malignancy, gastroesophageal reflux, migraine, renal failure, hypoalbuminemia, liver disease);

3) severe dehydration: weight loss >10% or standardized clinical dehydration score > = 18 for children aged 12-24 months and > = 16 for older children [20];

4) known hypersensitivity to ondansetron or domperidone;

5) previous enrolment in the study

### Intervention

After checking the inclusion and exclusion criteria, administration of oral rehydration solution (ORS) will be started following a standard protocol (Table 1). ORS will be prescribed by the doctor on call and administered under supervision of an emergency departments nurse. In case of failure of the initial ORS administration, defined as: 1) vomiting after ORS, assessed by the doctor on call or the emergency departments nurse based on the definition reported in Table 1; or 2) fluid refusal after three attempts), patients will be randomized to receive an oral administration of:

1) ondansetron syrup (0,15 mg/Kg of body weight);

2) domperidone syrup (0,5 mg/Kg of body weight);

3) placebo.

Children vomiting within 15 minutes after receiving the drug will be given a second dose. After 45 to 60 minutes from treatment administration, a new attempt to administer ORS will be done, according to the standard protocol (Table 1). After an adequate information of the study and before random allocation of subjects, written consent will be obtained by the doctor on call from the parents or legal guardian of children fulfilling the entry criteria and failing initial ORS administration. A register of all patients who were proposed to be enrolled in the study will be kept, and reason for refusal will be recorded.

**Figure 1:**
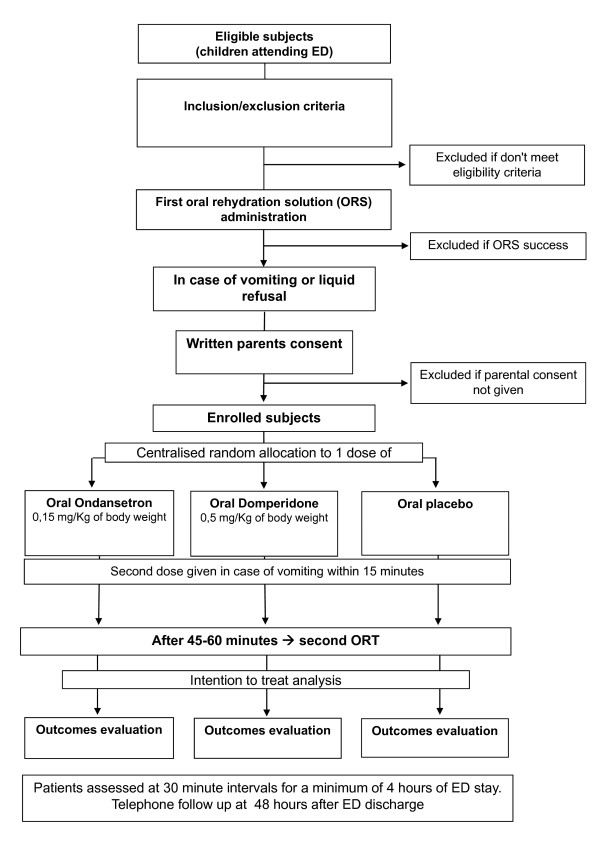
Study flow chart

A flow chart describing comparison groups and trial procedures is reported in Figure 1.

### Outcomes

#### Primary Outcome

Percentage of patients needing nasogastric or intravenous rehydration after symptomatic oral treatment failure, defined as vomiting or fluid refusal after the second attempt of ORT.

#### Secondary Outcomes

1) Percentage of subjects needing hospital admission for the same illness;

2) Percentage of subjects needing observation stay for more than 6 hours for the same illness;

3) Total emesis duration in the 3 allocation groups;

4) Number of episodes of vomiting in the 3 treatment groups during the follow-up period;

5) Percentage of subjects presenting adverse events.

Data regarding the study outcomes will be collected by doctors on call or bedside nurse during the patient stay in the ED (or any other paediatric unit devoted to short observation) using a standardized assessment tool. Patient will be reassessed at 30 minute intervals for a minimum of 6 hours. During the patients' hospital stay, doctors and nurses will be blind to treatment assignment. A telephone follow up call will be made 48 hours after ED discharge using a standard form by a research assistant blind to treatment assignment.

*Note: We discussed whether to consider hospital admission as the primary study outcome. Unfortunately, criteria for hospital admission could differ in different hospitals and ED settings and various administrative typologies of hospital admission exist in the study centres, including the "short observation" which may vary across centres in the way it is defined and applied. A recent systematic review on ondansetron confirms that when considering hospitalization as an outcome, there is great heterogeneity. Authors indicate different criteria for hospital admission as a possible explanation *[15]. *Definition and standardization of hospital admission criteria are difficult due to differences in the administrative and organizational characteristics among participating EDs. Furthermore, nasogastric or intravenous rehydration represent a more objective outcome and represent anyway a good proxy measure of hospital admission*.

### Randomization

Patients will be randomly assigned in fixed block size of nine to receive ondansetron or domperidone or placebo. Randomisation list will be generated using STATA software and will be stratified according to participating centres. Randomization procedure will be centralized and managed by an independent statistician at the Coordinating Centre. The randomization sequence will be provided to the central pharmacy, which will prepare and dispense to participating hospitals active drugs and placebo. After checking eligibility and failure of first ORS administration, the next available bag containing drug preparations will be opened by the doctor on call or by the bedside nurse and a weigh-appropriate dose will be administered to patients. All study investigators, personnel, and participants will be unaware of the randomization procedure and pharmaceutical preparations assignments.

### Blinding

The pharmaceutical preparation will be directly sent to participating centres in closed, opaque and consecutive numbered bags by the central pharmacy. Drug preparations will be indistinguishable by taste, odour and appearance. All study investigators, personnel, and participants will be blind to preparations administered.

### Information retrieval

A questionnaire detailing demographic data, medical history, allergies, history of present illness, and medication received will be collected at enrolment by doctors on call. Data relative to study outcomes will be collected by doctors on call or bedside nurse during the hospital stay. Patient will be reassessed at 30 minute intervals for a minimum of 6 hours and data will be collected at each assessment. Forty-eight hours after discharge, a blind researcher assistant will telephone the child's family to evaluate, using a standard form, the gastroenteritis evolution, the possible need of hospitalisation or readmission in ED and the final outcome. Paper records will be transferred by each centre into electronic data base. The percentage of subjects lost to follow up, based on available literature and taking into account the short follow up period, can be estimated as less than 10% [20,22].

### Sample size estimates

Studies comparing ondansetron versus placebo are available in the literature while it was impossible to identify studies comparing ondansetron and domperidone. Two studies evaluating domperidone versus placebo were also identified, even if characterized by low methodological quality and small sample size. Based on the available literature, it appears plausible to hypothesize that ondansetron is more efficacious in comparison to placebo or domperidone and therefore design the study as a superiority trial. For sample size estimation we specifically referred to the Roslund RCT [22] that implemented a similar protocol to ours (enrolment of subjects with clinical acute gastroenteritis who failed initial ORS administration in the ED). Taking into account the above stringent eligibility criteria, we estimate that the enrolment of 540 children (i.e. 180 patients in each arm) will provide the study with a statistical power of 80% to detect a change from 50% in placebo group to 35% in domperidone group and 20% in ondansetron group in the proportion of children requiring nasogastric or intravenous rehydration, given a two-sided type I error of 0,05. Given the lack of available efficacy estimates, domperidone efficacy was estimated as intermediate between ondansetron and placebo.

### Statistical analysis

Baseline characteristics of the three groups will be compared by the chi-square test for proportions and the analysis of variance or Kruskal-Wallis test (depending on data distribution) for continuous variables. Relative risks and 95% confidence intervals will be presented for categorical data while means and standard deviations for continuous data. For categorical outcomes, differences between groups will be evaluated using the chi-square test; for continuous outcomes using analysis of variance or Kruskal-Wallis test, depending on data distribution. Analyses will be performed with STATA software (version 9) according to the intention-to-treat principle. All p values will be two-sided, with a p value of less than 0.05 used to indicate statistical significance.

### Safety profile

An adverse event (AE) is any untoward medical occurrence in a patient or clinical investigation subject administered a pharmaceutical product and which does not necessarily have a causal relationship with this treatment. An AE can therefore be any unfavourable and unintended sign (including an abnormal laboratory finding, for example), symptom, or disease temporally associated with the use of a medicinal product, whether or not considered related to the medicinal product. All noxious and unintended responses to a medicinal product related to any dose should be considered adverse drug reactions (ADRs). The phrase "responses to a medicinal product" means that a causal relationship between a medicinal product and an AE is at least a reasonable possibility i.e. the relationship cannot be ruled out.

A serious AE (experience) (SAE) or reaction (SAR) is any untoward medical occurrence that at any dose:

- Results in death

- Is life-threatening event in which the patient was at risk of death at the time of the event; it does not refer to an event which hypothetically might have caused death if it were more severe

- Requires in-patient hospitalization or prolongation of existing hospitalization

- Results in persistent or significant disability/incapacity

- Is an important medical event

Medical and scientific judgment should be exercised in deciding whether expedited reporting is appropriate in cases of important medical events that may not be immediately life threatening or result in death or hospitalization but may jeopardize the patient or may require intervention to prevent one of the other outcomes listed in the definition above. These should also usually be considered serious.

In the event of the occurrence of any clinical AE or abnormal laboratory test value that is serious or medically important during the course of the study or the post-treatment period, irrespective of the treatment received by the patient, the investigator is obliged to immediately inform the Coordinating Units. Following a report by phone, written information has to be sent by fax or e-mail. Coordinating Units is solely responsible for sending the reports on Suspected Unexpected Serious Adverse Reactions (SUSARs) to all participating investigators, to AIFA and Ethics Committees concerned in accordance with international and Italian laws and regulations as well as ICH/GCP guidelines. SUSARs represent Serious Adverse Events related to study drugs, considered "unexpected" with regard to the Summary of Product Characteristics.

## Discussion

### Organizational Characteristics

The study will be coordinated by the Department of Paediatrics, Institute for Maternal and Child Health - IRCCS Burlo Garofolo, Trieste and by, Maternal and Child Health Laboratory, Institute Mario Negri, Milan. The Coordinating Units will be responsible for the trial coordination (including training, randomization, study monitoring, data collection, data analysis, reporting).

Subject will be enrolled in 15 Italian Paediatric Emergency Department Units.

A multidisciplinary (clinicians and epidemiologists) steering committee will establish to monitor the data, to ensure patient safety and to act as reference for Participants Units. Any problem arising during the trial will be discussed by committee members and decision will be shared with Participants Unit.

A period of 24 months will be required to carry out the study. The recruitment period will last 18 months. Each patient will be recruited at the ED of the participating centres and followed up every 30 minutes at least for 6 hours. Each patient will be contacted by phone 48 hours after ED discharge to evaluate long-term outcomes. Every 8 months research meetings between Coordinating and Participating Units will be arranged to discuss research development and address any emerging issue. The final report will be prepared within the 24 month period.

### Good clinical practices and quality control

Various processes will be undertaken in order to guarantee the overall quality of the study, and thus to maximise its validity and reliability:

1. The steering committee will monitor the safety and the overall quality and scientific integrity of the study. Any problems arising during the trial, including adverse events evaluation, will be discussed by the committee members and decision will be shared with Participants Unit. During the study a monitoring visit will be carried out in each centre by members of the steering committee;

2. Before the study starts and every 8 months, research meetings between Coordinating and Participating Units will be arranged to standardize the procedures, discuss research developments and address any emerging issues;

3. Local meeting with nurses and doctors involved in the field work will be arranged by local study coordinators to train, explain and discuss study procedures and flow charts;

4. Randomization procedure will be centralized and managed by an independent statistician at the Coordinating Centre.

5. The pharmaceutical preparation will be prepared and directly sent to participating centres by a central independent pharmacy.

6. All the documents produced for the study (Clinical research form, electronic database, informed consent) will be standardized;

7. Written documents on the trial process and a fieldwork manual will be prepared for doctors and nurses involved in the field work, including instructions on how to fill in the Clinical research form and the electronic database;

8. The electronic database will be periodically checked for accuracy by the Coordinating Units;

9. A progress report will be produced every eight months by the Coordinating Units.

## Abbreviations

AAP: American Academy of Pediatrics; AIFA: Agenzia Italiana del Farmaco (Italian Agency of Drugs); AG: Acute gastroenteritis; ARNO: ARNO project (ON-LINE drug prescription monitoring system - global vision); ED: emergency department; ESPGHAN: European Society for Paediatric Gastroenterology, Hepatology, and Nutrition; DRG: Diagnosis-related group; IRCCS: Istituto di Ricovero e Cura a Carattere Scientifico; IVT: Intravenous fluid therapy; NICE: National Institute for Clinical Excellence; NNT: Number Need to Treat; ORS: oral rehydration solution; ORT: oral rehydration therapy; RCT: randomised controlled trial; SUSARs: Suspected Unexpected Serious Adverse Reactions; WHO: World Health Organization

## Competing interests

This project has been funded by Italian Agency of Drugs, AIFA. The authors of this paper have no competing interests to declare.

## Authors' contributions

FM and LR conceived of and designed the study, obtained funding, and designed the intervention and evaluation procedures. MB, AC, AM, FR, DZ and GM, participated in the design of the study and the intervention and evaluation procedures. The remaining members of the Investigators group participated in the design of the study. All authors have read and approved of the manuscript.

## Pre-publication history

The pre-publication history for this paper can be accessed here:

http://www.biomedcentral.com/1471-2431/11/15/prepub
